# Finding Collaborators: Toward Interactive Discovery Tools for Research Network Systems

**DOI:** 10.2196/jmir.3444

**Published:** 2014-11-04

**Authors:** Charles D Borromeo, Titus K Schleyer, Michael J Becich, Harry Hochheiser

**Affiliations:** ^1^Department of Biomedical InformaticsUniversity of PittsburghPittsburgh, PAUnited States; ^2^Center for Biomedical InformaticsRegenstrief InstituteIndianapolis, INUnited States; ^3^School of MedicineIndiana UniversityIndianapolis, INUnited States; ^4^Intelligent Systems ProgramUniversity of PittsburghPittsburgh, PAUnited States

**Keywords:** translational medical research, cooperative behavior, interprofessional relations, interdisciplinary studies, information systems, information services

## Abstract

**Background:**

Research networking systems hold great promise for helping biomedical scientists identify collaborators with the expertise needed to build interdisciplinary teams. Although efforts to date have focused primarily on collecting and aggregating information, less attention has been paid to the design of end-user tools for using these collections to identify collaborators. To be effective, collaborator search tools must provide researchers with easy access to information relevant to their collaboration needs.

**Objective:**

The aim was to study user requirements and preferences for research networking system collaborator search tools and to design and evaluate a functional prototype.

**Methods:**

Paper prototypes exploring possible interface designs were presented to 18 participants in semistructured interviews aimed at eliciting collaborator search needs. Interview data were coded and analyzed to identify recurrent themes and related software requirements. Analysis results and elements from paper prototypes were used to design a Web-based prototype using the D3 JavaScript library and VIVO data. Preliminary usability studies asked 20 participants to use the tool and to provide feedback through semistructured interviews and completion of the System Usability Scale (SUS).

**Results:**

Initial interviews identified consensus regarding several novel requirements for collaborator search tools, including chronological display of publication and research funding information, the need for conjunctive keyword searches, and tools for tracking candidate collaborators. Participant responses were positive (SUS score: mean 76.4%, SD 13.9). Opportunities for improving the interface design were identified.

**Conclusions:**

Interactive, timeline-based displays that support comparison of researcher productivity in funding and publication have the potential to effectively support searching for collaborators. Further refinement and longitudinal studies may be needed to better understand the implications of collaborator search tools for researcher workflows.

##  Introduction

Building collaborative research teams is a critical challenge for biomedical scientists. Interdisciplinary research teams provide a breadth of expertise [1,2], shared workload [2,3], and greater advocacy for breakthroughs [2], often resulting in more frequent citations [4]. However, identifying appropriate collaborators is often difficult, particularly for junior investigators who lack extensive personal networks [5]. Research networking systems (RNSs) that model researcher activity, expertise, and collaborations have been developed to facilitate collaborator searches [6-9], particularly via federated search tools that provide preliminary demonstrations of cross-institution search facilities [10]. Emerging reports of RNS usage provide preliminary evidence of search and navigation patterns extracted from usage logs with deployed RNSs [11,12], but relatively little insight into how search tools should be designed to support the process of collaborator searches. The goal of this study was to conduct an iterative design and qualitative inquiry process to better understand scientists’ needs and workflows, and how they might best be supported by software tools. These efforts led to the development of a functional prototype collaborator search tool, which was evaluated in a preliminary usability study.

Identifying collaborators is a time-consuming process that does not scale well [6]. Researchers seeking collaborators often want to find new collaborators through existing contacts, who can provide useful feedback on the suitability of potential collaborators for their colleagues [6]. Although this approach might be effective for senior scientists with well-established personal contacts, junior researchers often lack personal contacts with potential collaborators [6]. Geographic separation is also a potential concern for evaluating potential collaborators, particularly given experience demonstrating the importance of physical proximity for research groups [13,14].

Identifying appropriate collaborators for team and translational science was one of the key motivations for the emergence of RNSs. As social networks for scientists, RNSs organize researchers’ interests, publications, funding, and collaborators in navigable formats designed to publicize research activity and support discovery of needed expertise. An assortment of commercial and academic RNSs provides a range of functionality, such as Digital Vita’s ability to populate National Institutes of Health (NIH) biosketches from RNS data [9]. Academic RNSs are typically deployed separately at individual research institutions [7], with localized navigation and search tools. Currently, one of the most prominent is the VIVO system [8], which provides a detailed semantic metadata model for describing researchers. Other notable tools include Harvard’s Profiles [15] and commercial tools, such as SciVal [16] and ResearchGate [17].

Concerns about the limitations of restricting searches to single institutions have led to the development of broader search tools. Direct2Experts uses a standard application programming interface convention to provide a federated multi-institution search interface [10]. Although Direct2Experts returns result counts that allow comparison across institutions, results are presented in their native form as provided by each institution. This lack of common formats limits opportunities for comparison and contrast. The VIVO platform’s use of semantic Resource Description Framework (RDF) markup and linked open data provides the possibility of cross-institutional searches, but this functionality is not well supported in current interfaces. The VIVO Searchlight browser plugin [18] demonstrates a possible approach to increasing the utility of RNS data by supporting links to individual VIVO profiles from multiple institutions through commonly used Web resources, such as PubMed entries [19,20].

Preliminary reports from institutional RNSs provide some insight into usage patterns and user goals. An analysis of 5 months of log data from an RNS at Columbia University found differences in usage patterns across user classes, with faculty performing more keyword searches than administrators [12]. A similar log-based analysis at the University of California, San Francisco, found that search engines were the source of almost 75% of initial visits, the number of return visitors increased over time, and that return visitors accessed a higher number of pages/visit compared to first-time users [11].

Relatively little attention has been paid to understanding how information tools might best support the process of searching for collaborators. Techniques such as contextual design [21] and scenario-based design [22] that rely on task modeling and work observation might be used to develop models of researcher goals, needs, and workflows, but the nature of collaborator search complicates these matters. As an occasional ad hoc task that generally lacks focused support from software tools, collaborator search use is not well suited for direct observation. This problem is particularly acute for RNS use. Given the incomplete penetrance of RNS systems [7] and a perceived lack of “critical mass” of participation for institutions where RNS systems have been deployed [23], ongoing use of these tools by researchers may be somewhat limited.

Preliminary investigations of user needs have identified some recurring themes in information needs and workflows. Schleyer et al [9] conducted retrospective interviews aimed at identifying researcher requirements for collaboration search tools, identifying themes such as compatibility of personal styles, rich communication needs including details beyond publications, high-quality data, and the importance of personal networks for the identification of collaborators. Bhavnani et al [24] conducted a qualitative study of researcher needs for tools for both collaboration identification and resource discovery, identifying the need for federated information, facilities for managing large volumes of information, and “humanized computing” tools that would favor user-controlled tools over algorithmic approaches that might use opaque processes to identify suggested resources. These suggestions are consistent with the observation from Boland et al [12] that different classes of RNS users may have different goals and workflows.

The goal of this study was to move beyond these descriptions of broad classes of user needs to explore specific features and designs, and to use these investigations to develop further understanding of user goals and preferences. Specifically, paper prototypes were used to elicit comments from researchers regarding their perceptions of preferences for interactive collaborative search tools. Qualitative analysis of responses to these prototypes was used to identify recurring requirements. These requirements informed the design of a functional prototype collaboration search tool, which was developed to provide preliminary evaluation of the feasibility and usability of interactive collaboration search tools. Results from these inquiries provided preliminary validation of the tool design while identifying areas of concerns that might need to be addressed in subsequent redesigns.

## Methods

### Summary

**Figure 1:**
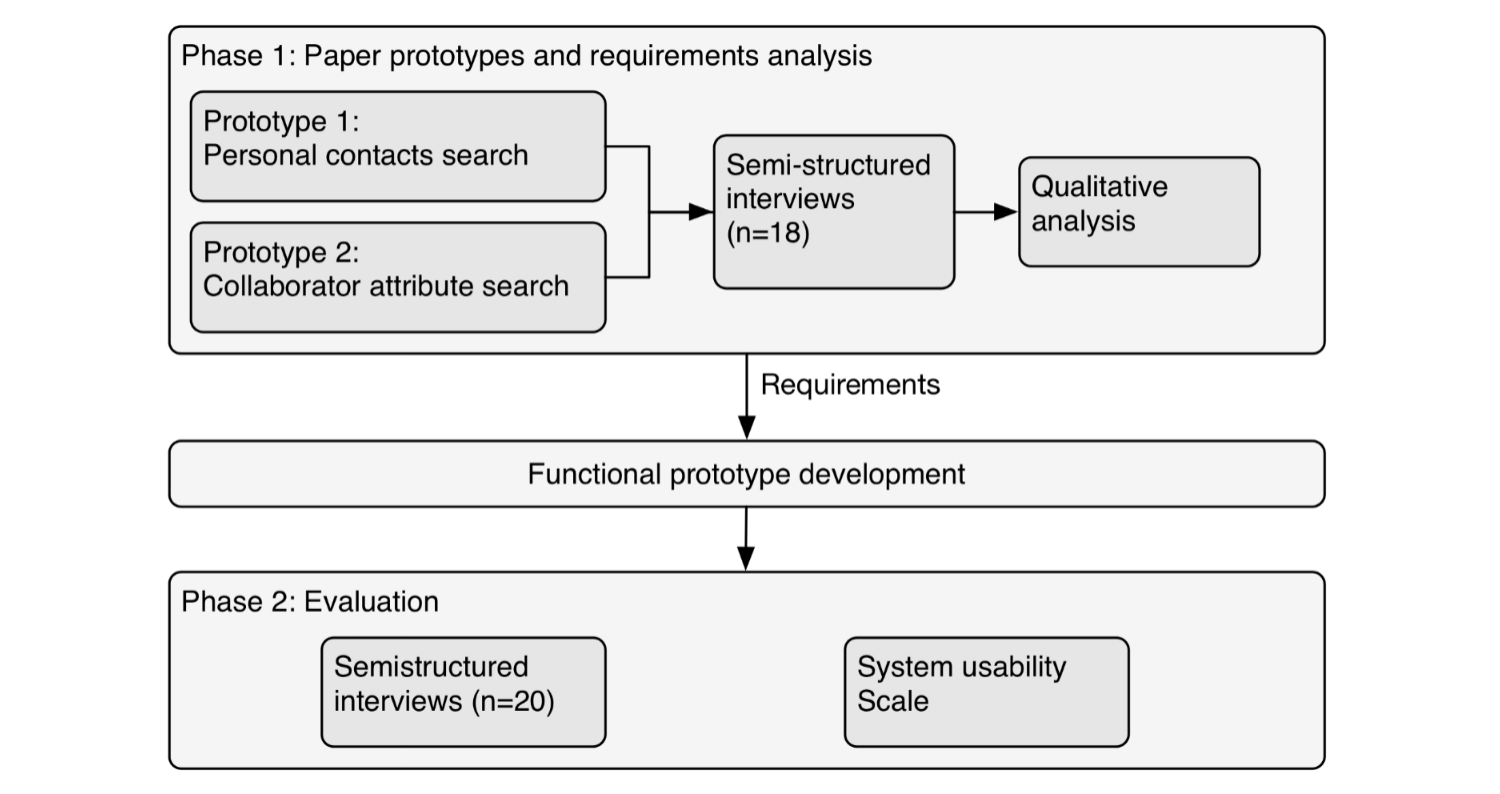
Overall study workflow.

This study used a combination of prototyping, qualitative inquiry, and software development. Initial designs of paper prototypes were based on findings from earlier studies [9]. Semistructured interviews with potential users [25] provided qualitative feedback, including reactions to the paper prototypes. These responses were analyzed to identify specific requirements, which were used to drive the design and implementation of a functional prototype. This prototype was evaluated through a second set of qualitative interviews with potential users (Figure 1).

### Paper Prototypes and Requirements Analysis

#### Overview

The goal of the first inquiry was to explore user requirements for collaboration search tools. Pilot studies presented a conundrum: potential users were likely to be unfamiliar with the notion of collaborator search tools because of the relatively low adoption rates of RNSs. To effectively elicit participant input, we developed 2 paper prototypes illustrating hypothetical interfaces for collaborator search tools. We use the term “paper” here to informally refer to low-fidelity, nonfunctional prototypes. Using multiple prototypes provided the freedom to consider designs that covered a variety of perspectives on relevant information and to present participants with a range of options that might elicit more detailed feedback [26].

#### Prototype 1: Personal Contacts Search

**Figure 2:**
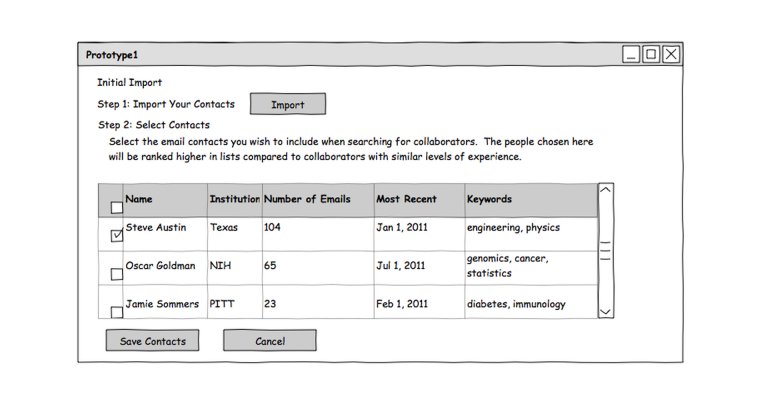
Personal contacts search: contact import screen. This screen allows users to import existing contacts from an external source (eg, email) and these contacts are then matched against publication data from the RNS.

Researchers often seek new collaborators through existing contacts [6]. The first prototype explored the possibility of using prior contacts from an external source such as an email contact list to begin a collaborator search (Figure 2). These contacts would then be matched to publications and author information found within an RNS.

Use of this tool begins with importing email contacts. Users then use keyword searches to explore topics of interest. These keyword searches leverage RNS publication and grant data, identifying possible collaborators who have relevant publications. Potential matches are listed in rows on the screen. Information about each candidate is arranged in chronological order along a horizontal timeline. Publications are marked with color codes to indicate individuals who are on the imported contact list, geographically close (within 10 miles of the user), and/or marked as interest for further follow-up. Checkbox filter selections can be used to filter out items based on any of the color-coded categories.

**Figure 3:**
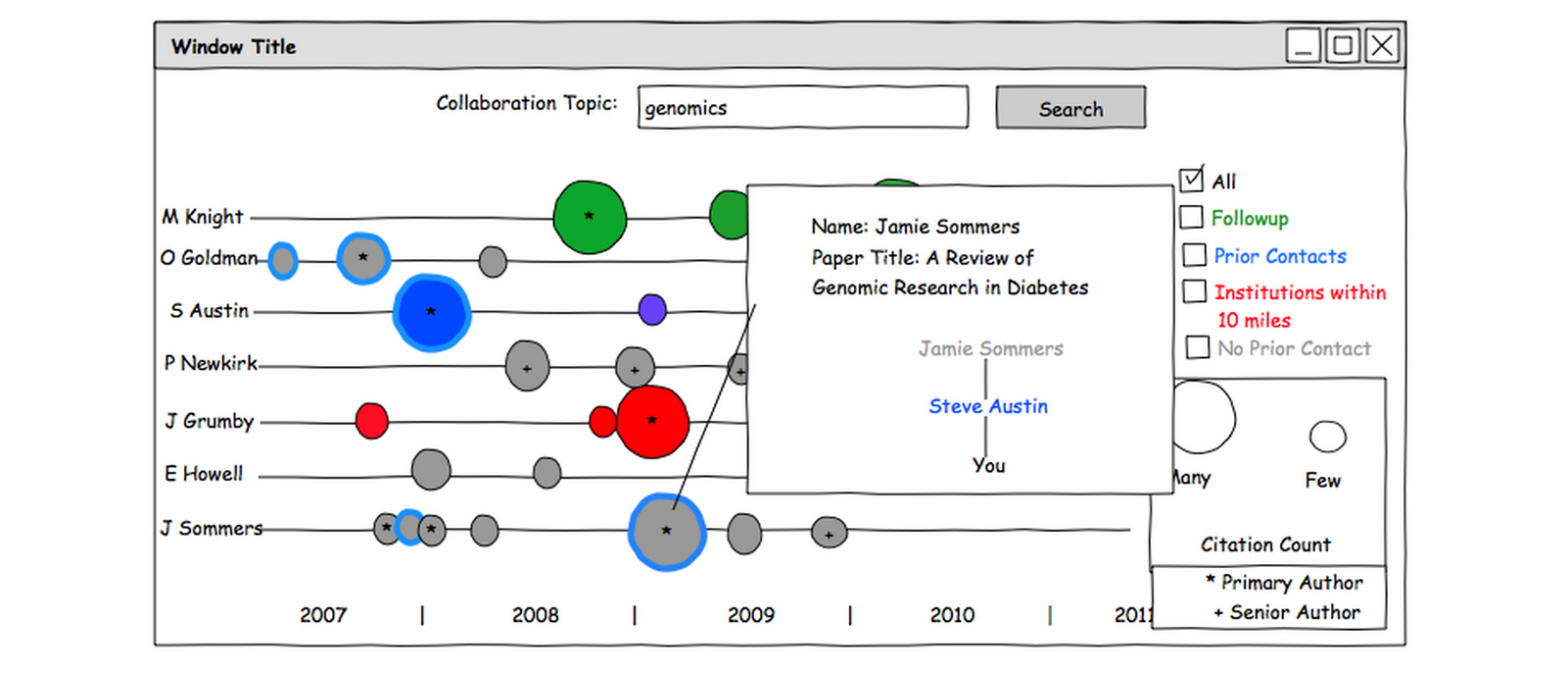
Personal contacts search results. This screen shows a list of collaborators who have published on the topic “genomics”. Their publications are color-coded: red indicates institutions within 10 miles of the user, blue means the author was on the user’s contact list, green means the user has marked the collaborator for future contact, and gray is the default color. Circles can be coded with blended colors to indicate multiple categories. Thus, purple indicates nearby (red) authors on the contact list (blue). The circles are sized to indicate the number of citations. The user has selected a paper and their relationship with the author is displayed.

For candidates not found on the user’s contact list, coauthorship information can be used to identify current contacts who might have coauthored papers with them (Figure 3).

#### Prototype 2: Collaborator Attribute Search

Seniority can play a major role in collaborator search: junior researchers often seek junior collaborators, perhaps because more senior researchers often decline collaboration requests [5,24]. The second prototype uses a 2-step approach to support the use of seniority in identifying candidates. The use of this tool begins with the identification of a potential collaborator in an RNS, perhaps through browsing lists of participants. The data for this individual is used to formulate a “profile” for subsequent searches, quantifying different aspects of a researcher’s history (eg, overall number of publications, grant funding) into measures that will be used for subsequent comparisons against other candidates. The user can then search the system for a topic of interest based on research keywords similar to those used in the first prototype.

**Figure 4:**
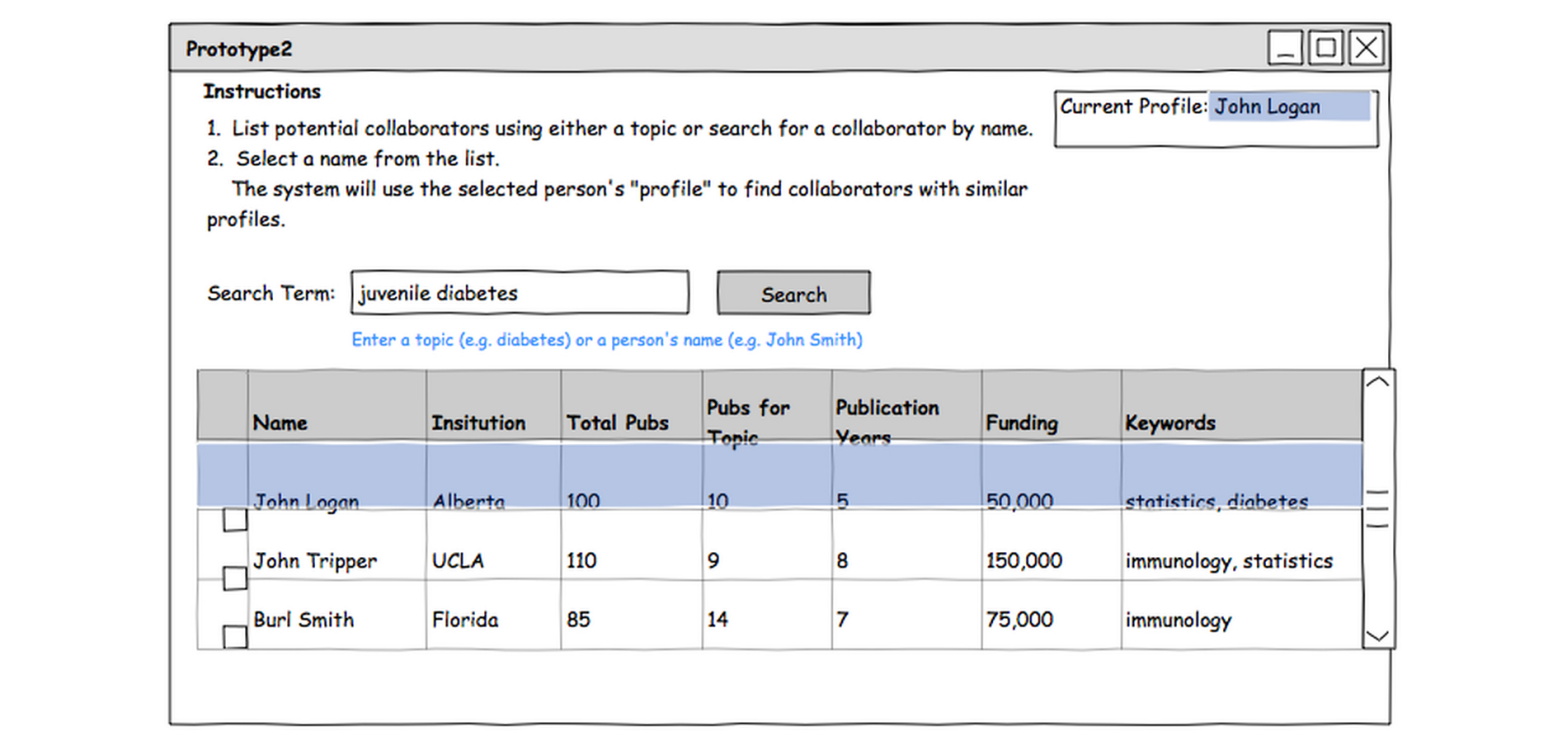
Collaborator attribute search: selecting a profile. A selected profile (“John Logan”) forms the basis for a similarity search (“juvenile diabetes”) that constrains the candidates returned by subsequent keyword queries. Selecting the profile of a junior researcher might bias results of subsequent searches toward junior researchers.

Similar profiles are then computed for each candidate returned by the topic search and compared to the selected profile. The candidates who are most similar to the selected profile are shown on the screen. Thus, initial selection of a profile of a junior researcher might bias subsequent results to favor other junior researchers (Figure 4).

**Figure 5:**
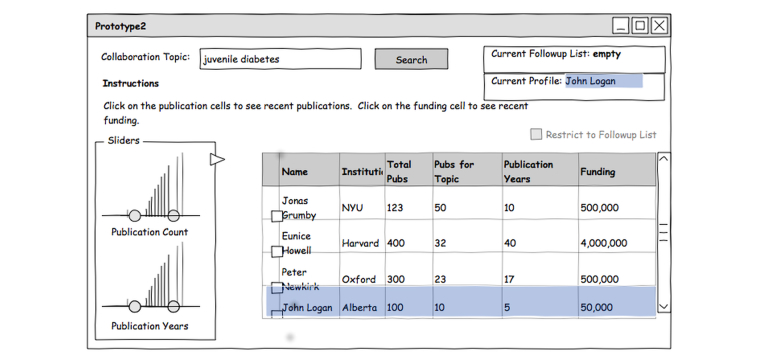
Collaborator attribute search with dynamic filters. The sliders on the publication counts and years are double-sided allowing researchers to restrict the criteria in either direction. The sliders can adjust publication counts, publication years, funding, and number of publications related to the chosen topic (juvenile diabetes) Histograms on the sliders display distribution of the possible values across items in the currently active set [27].

Search results are shown in a table containing researchers’ names, institutions, total number of publications, number of publications matching the search term, the number of years of active publication (a proxy for seniority), an estimate of total research funding (based on grant information), and keywords summarizing their primary research interests. Interactive double-thumb sliders provide the ability to set upper and lower bounds on the attributes in the table (Figure 5) with histograms on the slider providing a display of the distribution of the given values across the currently active candidate profiles [27].

This prototype also differs from the first (Figure 3) in terms of both information provided and the representation of that information. Where the first prototype provides chronologically oriented feedback in graphical form along with contact-based information and geographic hints, the second provides tabular aggregate data. The collaborator attribute search prototype also provides affiliation information and additional matching keywords not available in the personal attributes search design. A summary of key features of the 2 prototypes is given in Table 1.

These prototypes were used to elicit feedback from potential users, including both general preferences for collaborator search tools and specific responses to specific design features. Participant sessions consisted of a structured interview and unstructured discussion of the prototypes. The structured interview included questions concerning demographics, social networking applications usage, and workflows for finding collaborators (interview questions are given in Multimedia Appendix 1). Participants were asked to respond to all questions that they felt were applicable to their work. The interviewer then described and presented each of the prototypes to the participants, using several screens that simulated possible uses of each system. Participants were asked to identify features of the prototypes that they thought would be particularly useful, to note features that appeared to be less worthwhile, and to describe new features that they might like to see added. Finally, they were asked to provide overall impressions, considering both of the prototypes. Each participant saw both of the prototypes with the order of presentation of the prototypes varied between participants.

Sessions were conducted online using the WebEx Web conferencing tool [28], which was used to present the prototype screens to the participants and to record the screenshots and audio from the sessions. Descriptive statistics were used to characterize participant background, education, and collaborator search behavior. Audio and screen capture recordings of the sessions were analyzed and coded using an open-coding approach [25,29]. Specifically, 1 author (CB) reviewed the audio recordings using descriptive codes to classify participant comments including reactions to the prototypes, statements about collaboration finding practices, preferences/requirements for collaboration finding software, etc. Initial codes were chosen based on content of the interactions and eventually categorized as patterns emerged. Higher-level themes identified during this process formed the basis for categorizing requirements for the functional prototypes. A second author (HH) reviewed all codes and categorization. This study was classified as exempt by the University of Pittsburgh Institutional Review Board, Study #PRO12060527.

**Table 1:** Feature comparison of both Phase 1 prototypes.

Functionality	Prototype 1: personal contacts search	Prototype 2: collaborator attribute search
Search mechanism	Keyword search and link to imported contacts	Browse/search for initial profile, keyword search identifies researchers with similar profiles
Display	Timeline with color-coded glyphs for publications	Tabular grids with aggregate displays of publications, grant funding, institutions, and other keywords
Controls	None	Interactive controls for selecting similarity values for publications, grants, and other values

### Functional Prototype Development

**Figure 6:**
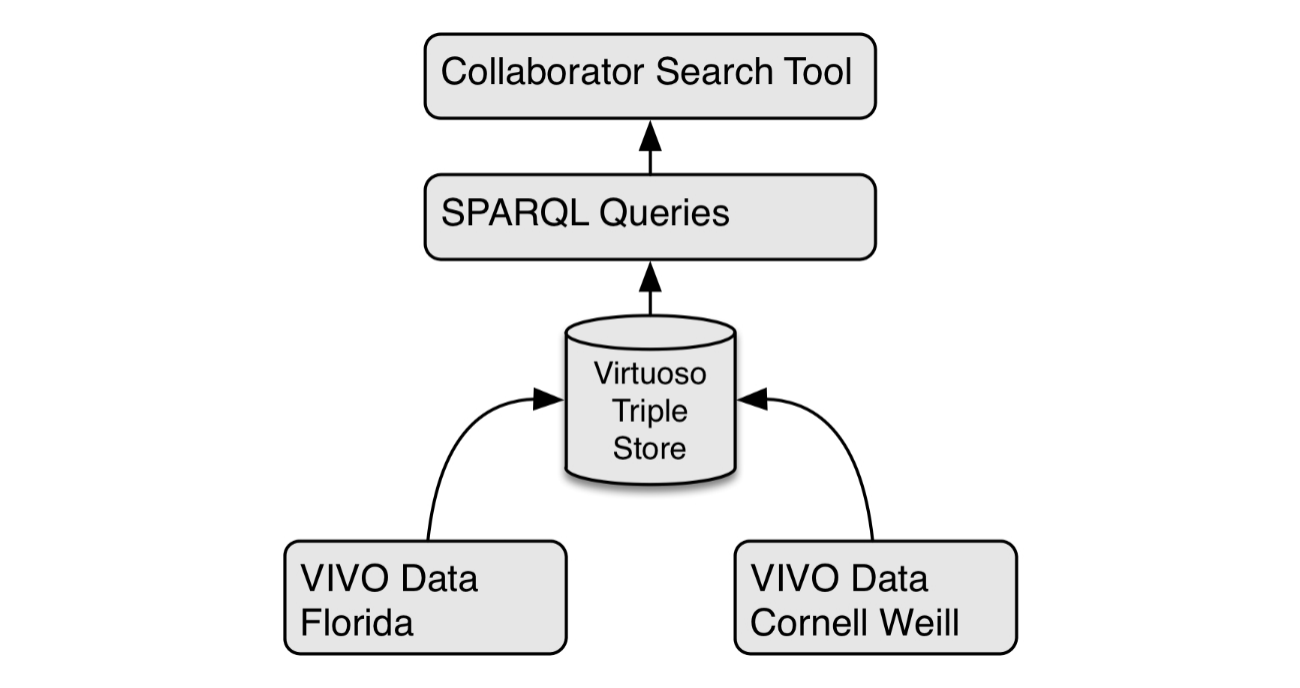
Prototype architecture.

Although paper prototypes can provide useful formative feedback for workflow and interface designs, static representations may fail to convey the dynamic nature of interactive tools. A functional prototype was implemented to provide a working example of a tool designed to satisfy the requirements derived from the initial qualitative inquiries. A Virtuoso Open-Source Edition triple store [30] was used to store RDF-formatted VIVO [8] data from the University of Florida and Weill Cornell Medical College. Data from the triple store was retrieved through SPARQL Protocol and RDF Query Language (SPARQL) [31] queries. The Web-based prototype was developed using the D3 library [32], which uses scalable vector graphics and JavaScript to create interactive data visualizations. JavaScript code developed for the prototype issued SPARQL queries against the Virtuoso triple store, passing the results to the D3 library for visualization. The system architecture is illustrated in Figure 6.

### Functional Prototype Evaluation

Evaluation of the functional prototype involved asking participants to use the tool to conduct collaborator search tasks. Each participant session began with a series of questions similar to those used in Phase 1 (see Multimedia Appendix 2 for all questions). The participant then completed 2 collaborator search tasks, 1 using the prototype and the other using their choice of online search engines and repositories such as PubMed. Because the alternative online tools did not provide a directly comparable experience, they were used only to provide contrast to the prototype tool and we do not discuss these interactions here. One task asked participants to find collaborators familiar with Alzheimer’s disease, the other specified researchers in Parkinson’s disease. These were chosen to be fairly broad to avoid dependence on user expertise and to minimize risk of bias associated with participant familiarity with the research field. The order of both tasks and tools was varied across participants.

After completing the tasks, participants were interviewed regarding the impressions of the prototype. Participant responses to the tool were evaluated using the System Usability Scale (SUS) [33,34]. Additional Likert scale questions asked participants to respond to key features of the prototype on a Likert scale (1-5, 5 being best). Interviews were conducted via WebEx and demographic and search behavior data were analyzed as in the earlier phase of the study.

## Results

### Paper Prototypes and Requirements Analysis

#### Overview

Study participants represented 11 US-based research institutions and 1 European university. Participants included both principal investigators (PIs) and research facilitators (RFs)—members of institutional research offices and others who assist investigators in forming project teams. Most participants in both categories had doctoral degrees (Table 2).

**Table 2:** Demographics of study participants from the first (N=18) and second (N=20) studies according to research role, education, and gender.

Participant classification			Paper prototype participants (N=18)	Functional prototype participants (N=20)	Total (N=38)
**Research role and degree**					
	**Principal investigators**				
		BS/MS	5	3	8
		PhD	6	9	15
		PharmD	0	0	0
		MD	1	1	2
		MD/PhD	0	2	2
		Total	12	15	27
	**Research facilitators**				
		BS/MS	3	3	6
		PhD	3	0	3
		PharmD	0	1	1
		MD	0	1	1
		MD/PhD	0	0	0
		Total	6	5	11
**Gender**					
	Male		5	11	16
	Female		13	9	22

Responses to structured interview questions regarding collaboration behavior indicated that participants were active researchers with a range of strategies for identifying collaborators. Most respondents (12/18, 66.7% in the paper prototype sessions; 19/20, 95% in the functional prototype sessions) answered all questions completely. Respondents participated in a mean of 5.4 (SD 6.3) research projects in the previous 12 months, interacting with a mean of 31.9 (SD 29.4) unique collaborators. Participants were active collaborators, having been approached with a mean of 5.2 (SD 5.3) offers of collaboration during the 12 preceding months, and having contacted others a mean of 6.7 (SD 13.9) times on average during the same interval (Table 3).

The approach to finding collaborators most frequently cited was using existing social networks, selected by 72% (13/18) of respondents. Participants who used software tools reported using NIH resources, homegrown systems, a variety of commercial social networking tools, and search engines (Table 4).

Coding of responses to the paper prototypes led to the identification of several emergent themes, which were used to derive requirements. Specifically, we identified 3 key themes: measuring research productivity, tracking candidates identified as potential collaborators, and conducting complex searches. Specific requirements addressing each of these themes were also identified (Table 5).

**Table 3:** Participation in collaborations.^a^

Question		Prototype, mean (SD)		Overall, mean (SD)
		Paper (n=18)	Functional (n=20)	(n=38)
**2. Approximately, how many funded research projects have you participated in during the past 12 months?**				
	Principal investigators	3.2 (1.8)	4.9 (3.8)	4.2 (3.2)
	Research facilitators	10.3 (13.9)	7.8 (9.1)	8.9 (10.8)
	Overall	5.1 (7.4)	5.6 (5.5)	5.4 (6.3)
**3. Approximately, what was the average number of collaborators you directly interact with in each project?**				
	Principal investigators	18.5 (21.9)	13.2 (15.9)	15.6 (18.5)
	Research facilitators	5.5 (0.7)	20.4 (33.4)	16.1 (28.3)
	Overall	16.5 (20.6)	15.1 (21.0)	15.7 (20.5)
**4. What is the total number of unique collaborators you have interacted with in the last 12 months?**				
	Principal investigators	27.1 (26.8)	29.0 (19.9)	28.3 (22.2)
	Research facilitators	15.0 (7.1)	56.2 (51.8)	44.4 (46.9)
	Overall	24.9 (24.6)	35.8 (31.7)	31.9 (29.4)
**5. During the past 12 months, how many times have you been approached for a formal collaboration?**				
	Principal investigators	5.9 (3.6)	5.2 (2.9)	5.5 (3.1)
	Research facilitators	1.0 (1.4)	5.6 (3.7)	4.3 (3.8)
	Overall	5.1 (3.8)	5.3 (3.0)	5.2 (3.3)
**6. During the past 12 months, how many times have you contacted someone about a potential collaboration?**				
	Principal investigators	5.1 (3.0)	3.1 (2.5)	3.9 (2.8)
	Research facilitators	11.0 (12.7)	19.0 (34.1)	16.7 (28.6)
	Overall	6.1 (5.2)	7.1 (17.3)	6.7 (13.9)

^a^Question 1 results reported in Table 2.

**Table 4:** Tools used to find collaborations. Multiple responses for each question were allowed.^a^

Question		Prototype, n (%)		Overall, n (%)
		Paper (n=18)	Functional (n=20)	n=38
**7. How do you usually find collaborators?**				
	Existing network	13 (72)	16 (75)	29 (76)
	NIH resources	5 (28)	4 (20)	9 (24)
	Research networking system	3 (17)	1 (5)	4 (11)
**8. Are there specific tools you use to find collaborators (eg, PubMed, NIH Reporter, Web of Science)?**				
	NIH Resources	4 (22)	6 (30)	10 (26)
	Homegrown System	4 (22)	2 (10)	6 (16)
	Web of Science	3 (17)	1 (5)	4 (11)
	SciVal	2 (11)	2 (10)	4 (11)
	LinkedIn	2 (11)	0 (0)	2 (5)
	Google	3 (17)	0 (0)	3 (8)
	Community of Science	1 (6)	1 (5)	2 (5)
**9. Do you use general purpose networking applications for professional purposes (eg, FaceBook, LinkedIn, Google+)?**				
	LinkedIn	5 (28)	7 (35)	12 (32)
	Facebook	4 (22)	0 (0)	4 (11)
	Google+	1 (6)	2 (10)	3 (8)
	Twitter	1 (6)	2 (10)	3 (8)
	Friendfeed	0 (0)	1 (5)	1 (3)
**10. Do you use a scientific collaboration tool (e,g., VIVO, CAP, Loki, ResearchGate)?**				
	SciVal	4 (22)	5 (25)	9 (24)
	VIVO	1 (6)	2 (10)	3 (8)
	Digital Vita	0 (0)	1 (5)	1 (3)
	Leo	1 (0)	0 (0)	1 (3)

^a^Users were allowed to select all appropriate values for each question.

**Table 5:** Themes derived from the paper prototypes mapped to the requirements used to design the prototype.

Theme and information needed		Associated requirement
**Measure impact**		
	Which candidates are productive researchers?	1. Chronological display of grants and publications
	Who are the leaders in the field?	1. Chronological display of grants and publications
	Which candidates have had the most impact?	2. Robust impact measures
**Track candidates**		
	Which individuals are under consideration?	3. Tools for tracking promising candidates
	What additional information might be gathered about each candidate?	3. Tools for tracking promising candidates
**Conduct complex searches**		
	Which candidates work in multiple fields?	4. Multiple keyword search

#### Requirement 1: Chronological Display Data of Grants and Publications

The first prototype included a page that displayed publications on a horizontal timeline (Figure 3). Participants found this screen to be helpful when searching for collaborators, facilitating both the comparison of candidates on the basis of the frequency and timeliness of their publication on topics of interest, and the identification of key leaders in specific subfields. Simultaneous presentation of both publications and grants was suggested as a possible improvement on the prototype designs.

#### Requirement 2: Robust Impact Measures

Although participants were interested in seeing quantitative measures of research impact, there was no agreement on specific measures. Citation counts, h-index [35], and journal impact factors were discussed, but there was no consensus as to which would be preferred.

#### Requirement 3: Tools for Bookmarking Promising Candidates

Both prototypes allowed users to maintain lists of potential collaborators. Participants were enthusiastic about this feature. In addition to a list, participants suggested allowing users to annotate candidates with free-text notes commenting on reasons for selection of each individual or other reminders relevant to the collaborator search tasks.

#### Requirement 4: Multiple Keyword Search

Participants thought the single keyword presented in both prototypes was too limiting. Several ideas were suggested, including the addition of sliders to adjust the “weight” of keywords in a search, displaying primary/secondary keywords, and including additional keywords in searches.

Participants did not respond well to some proposed features. Contact lists were not viewed as being particularly useful. Some respondents questioned the utility of seeing existing contacts in a search tool because they already know who their contacts are; therefore, seeing them listed in the tool would not be helpful. Participants were ambivalent regarding the physical distance between the participants and their potential collaborators. Some of the participants said this was useful information whereas others said this information was not necessary. Comparison of responses from the 2 groups of users (PIs vs RFs) did not reveal any systematic differences between the groups in responses to the prototypes or requirements for collaborator search tools.

### Functional Prototype Development

The interactive prototype implemented most of the requirements identified from analysis of the paper prototype data (Table 6). Requirement 2 (robust impact measures) was not implemented in the prototype because participants were not able to agree on a single method for ranking authors based on bibliometric measures.

**Figure 7:**
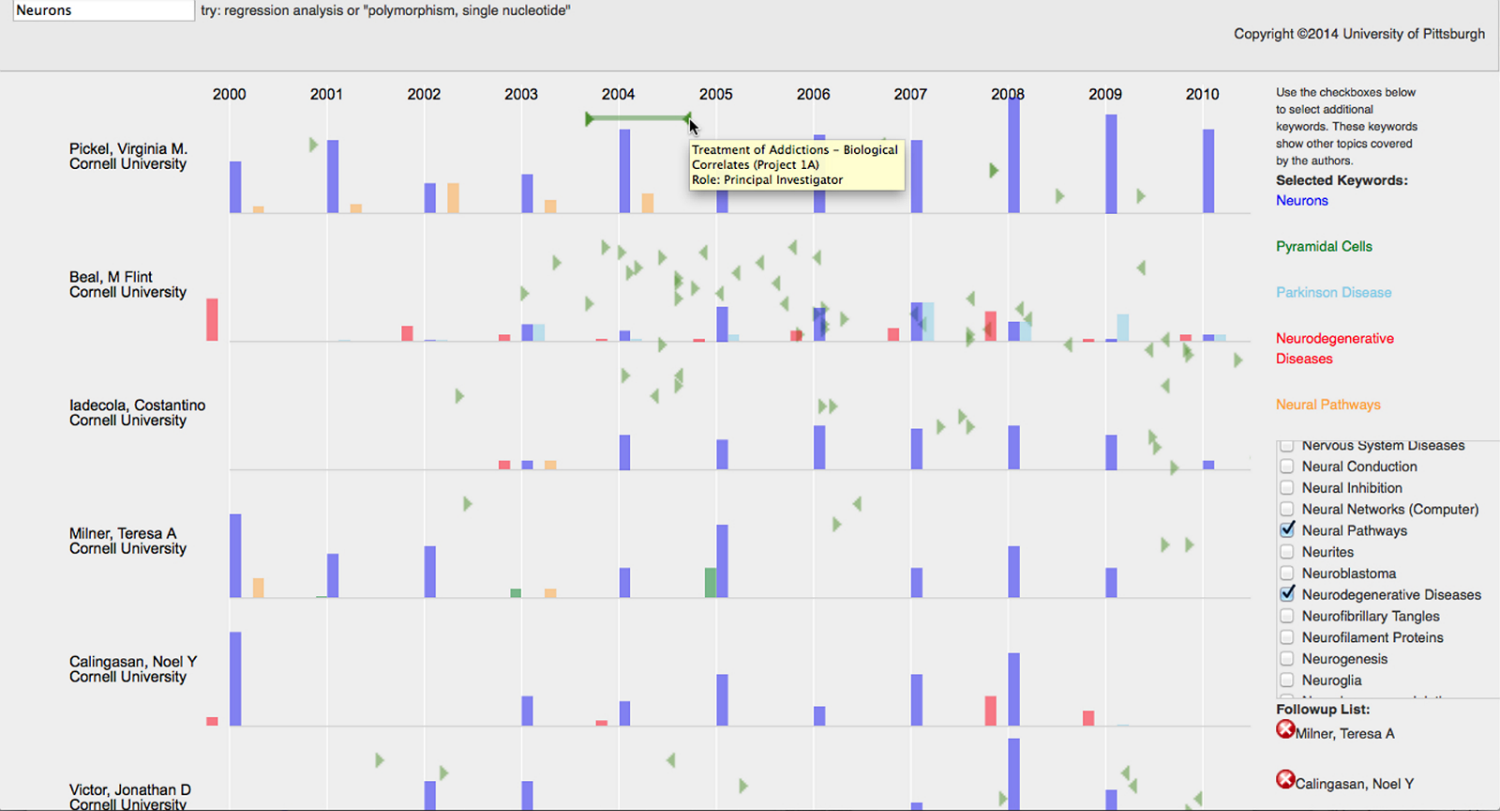
Screenshot of prototype. An initial search for the term “neurons” has been augmented by selection of additional terms (“pyramidal cells”, “Parkinson disease”, “neurodegenerative diseases”, and “neural pathways”) from the list of terms associated with neurons (right column). The rectangles represent the frequency of categorized publications, color-coded by each MeSH term. Green triangles represent the start/end dates for a collaborator’s grants. When the user mouses over an endpoint of a grant, a line is drawn showing the grant’s full extent and a tooltip describing the grant is also shown. Researchers of interest can be added to the follow-up list (lower right) by clicking on the name and adding a descriptive comment.

The prototype is based on a timeline-based view of candidates’ publication and grant history. The main view displays potential candidates’ names and institutions on the y-axis with each candidate’s grants and publications displayed in a horizontal line on the x-axis. The prototype employs distinct visuals for the grant and publication data. Pairs of green triangles are arranged on the timeline representing the start and end dates of the grants. Rectangles act as bar charts summarizing a candidate’s publications for a year. Keyword filters and a “bookmark” list of candidates identified for subsequent follow-up can be found on the right hand side of the screen (Figure 7).

To find collaboration candidates, the user types a keyword into the search box in the upper left corner (Figure 7). Autocomplete functionality matches user input against the list of all Medical Subject Headings (MeSH) topics from the publications found in the VIVO data. When a search term is selected, the prototype retrieves all candidates who have published articles associated with that term. Candidates are displayed in rows with each row displaying a timeline of the individual’s grants and annual publication counts (since 2000). Publications are indicated as bars, providing a histogram of publication counts categorized and color-coded by keyword for each year.

The height of each bar corresponds to the percentage of the candidates’ publications from a given year that correspond with the associated keyword. For example, given a user search for “neurons”, a candidate who had 6 of 10 publications for a given year matching that term would have a bar for that year that occupied 60% of the maximum possible height. Because mapping MeSH keywords to grant topics is not straightforward [36], all articles for each candidate are displayed even if they do not match the search term(s). Grants are displayed on the timeline as bracketed green arrows indicating the start and end of each grant. This design was chosen as being less cluttered than alternatives that drew lines for the complete duration of the grant.

The selection of a search term also generates the display of a list of additional keywords in a set of checkboxes on the right of the screen. The additional keywords represent the superset of all the terms in the publications retrieved during the initial keyword search. The keywords are arranged alphabetically and color-coded up to a maximum of 4 additional keywords. Clicking the checkbox for additional keywords creates new publication bars corresponding to those keywords providing an opportunity to visualize research activity in multiple areas, thus satisfying requirement 4 (multiple keyword search).

The prototype contains several interactive features. Hovering over the candidate’s name lists their top 8 publication topics and a research overview (if available from the VIVO datasets). Hovering over the publication bars leads to a display of the titles of the publications for a given color-coded keyword plus the summary (eg, 8 of 10 papers for 2012). To interact with grant information, users can move the mouse between the start and end date for a grant. When this happens, a green line connecting the start and end of the grant is displayed. A box above this line displays the title of the grant plus the candidate’s role in the grant (eg, principal investigator, coinvestigator). The dates on the x-axis and the green line allow the user to easily see the duration of the grant.

Users can put promising candidates on a bookmark list. To add a candidate to the bookmark list, the user clicks on a candidate’s name and optionally enters a note describing why the candidate was selected. The bookmark list is shown on the lower right side of the prototype. Source code for the prototype is available in a GitHub source code repository [37].

**Table 6:** Requirements mapped to prototype functionality included in the working prototype.

Requirement	Prototype feature(s)
1. Chronological display of grants and publications	X-axis used as a research history timeline
	Publications shown as rectangles
	Grants shown as triangles
2. Robust impact measures	Not implemented
3. Tools for tracking promising candidates	Candidate names can be added to a bookmark list shown alongside timelines
	Users can record notes about candidates
4. Multiple keyword search	Users search for 1 keyword
	Checkboxes allow users to add up to 4 additional keywords

### Functional Prototype Evaluation

A total of 20 participants from 12 US-based institutions were recruited to evaluate the functional prototype (Table 2). Similar to the first phase of the study, recruitment included individuals involved in biomedical research. Participants were comparable to participants from the earlier inquiry in participation in funded projects and collaborator search patterns (Tables 3 and 4).

The participants evaluated the prototype using the SUS [33] (Table 7). The SUS employs a set of 10 Likert-type questions. The overall test is set on a scale from 0-100 (where 100 is represents the best possible score). The mean SUS score for all participants was 76.4 (SD 13.9). For PIs, the mean was 77.5 (SD 14.0), and the mean for RFs was 73.0 (SD 14.3). According to Bangor [34], a score greater than 70 represents a passable system from a usability standpoint. Although small sample sizes (n=5 for RFs) prevent statistical comparison, results across the 2 participant groups seem comparable. Examination of responses to individual questions revealed a mixed picture: although respondents generally agreed that the system would be learnable, responses to other questions suggested potential concerns regarding topics such as complexity, anticipated frequency of use, inconsistency, and the need for support in using the system (Table 7).

Additional Likert scale questions rated functionality of the prototype. On a scale of 1 to 5, with 5 indicating a most useful feature, all features (except 1) scored greater than 4 (Table 8).

**Table 7:** Functional prototype usability results: mean and standard deviations for the individual SUS questions used in the evaluation phase (1=strongly disagree, 5=strongly agree).

SUS question	PIs, mean (SD) (n=15)	RFs, mean (SD) (n=5)	Overall, mean (SD) (n=20)
I think that I would like to use this system frequently	2.9 (1.5)	3.6 (0.55)	3.1 (1.4)
I found the system unnecessarily complex	4.3 (1.0)	4.4 (0.89)	4.4 (0.99)
I thought the system was easy to use	4.3 (0.60)	4.3 (0.84)	4.3 (0.66)
I think that I would need the support of a technical person to be able to use this system	4.7 (0.60)	4.0 (1.1)	4.6 (0.83)
I found the various functions in this system to be well integrated	3.5 (0.76)	3.4 (0.81)	3.5 (0.76)
I thought there was too much inconsistency in this system	4.3 (0.87)	4.0 (1.0)	4.3 (0.86)
I would imagine that most people would learn to use this system very quickly	4.2 (0.66)	4.2 (0.45)	4.2 (0.62)
I found the system very cumbersome to use	4.1 (1.1)	4.0 (1.2)	4.2 (1.0)
I felt very confident using the system	3.9 (1.1)	3.6 (0.55)	3.9 (0.99)
I needed to learn a lot of things before I could get going with this system	4.3 (1.1)	3.8 (1.6)	4.2 (1.1)

**Table 8:** Mean and standard deviations of scores for Likert questions^a^ regarding features within the prototype.

System functionality	PIs, mean (SD) (n=15)	RFs, mean (SD) (n=5)	Overall, mean (SD) (n=20)
Overall timeline format	4.7 (0.59)	4.4 (0.89)	4.65 (0.67)
Height of the publication bar	2.8 (0.94)	3.2 (0.83)	2.90 (0.91)
Document data available on hover	4.4 (0.91)	4.2 (0.45)	4.35 (0.81)
Grant title and role available on hover	4.8 (0.41)	4.0 (0.0)	4.60 (0.50)
Grant start and end dates indicated on timeline	4.7 (0.62)	3.0 (0.0)	4.25 (0.91)
Ability to add/remove collaborators to a list	3.9 (1.1)	4.4 (0.89)	4.05 (1.1)
Ability to add notes regarding candidates	3.9 (1.2)	4.4 (0.89)	4.05 (1.1)
Ability to add/remove additional keywords	4.3 (0.88)	3.8 (0.83)	4.2 (0.88)

^a^On a 5-point scale (1= useless, 5=useful).

Overall, the participant reaction to the working prototype was positive. The combination of publication and grant information in a single timeline scored the highest (mean 4.65, SD 0.67) of all the prototype features. Participants felt the interface provided insights into the candidate’s research interests and past history. The timeline format allowed users to examine researchers’ publication history, including both the recency and frequency of publications associated with search keywords. The publications allowed users to categorize a candidate as either a multidisciplinary researcher or a researcher with a single field of research. As with the SUS scores, responses from the 2 groups are roughly similar.

Several improvements to the application were suggested during the evaluations. Multiple participants requested hyperlinks to the PubMed and NIH Reporter records corresponding to items found in collaborator profiles. One evaluator felt that information regarding candidates’ academic training (eg, MD or PhD) was important for assembling collaborative teams. Another evaluator observed that papers and grants do not fully describe the value a candidate brings to collaborations, particularly including unique expertise or access to crucial resources (eg, animal models, computing techniques). The addition of research resource information [38-40] was suggested as a potential solution to this problem.

Anecdotal feedback from participants suggested that interest in collaborator search tools might differ based on the context in which researchers work. Two participants—1 from a country with a smaller number of universities and another from a small US medical school (less than 200 faculty)—commented that the lack of resources at their institutions limited opportunities for local collaborations, potentially motivating greater interest in RNS tools.

## Discussion

### Principal Findings

Interactive visualizations may help researchers use RNSs to identify collaborators. Interviews with researchers used paper prototypes to stimulate discussion of desired functionality for collaborator search tools. A functional prototype providing many of these features, including chronological displays, bookmarking tools, and multiple keyword search, was well received by users. Additional development and evaluation will be needed to gauge the utility of RNS collaborator search tools.

### Building Usable Collaborator Search Tools

Identifying appropriate collaborators is an important task in the increasingly interdisciplinary field of biomedical research. Although RNSs show great promise for aggregating and representing data describing researchers and their potential contributions, the success of these tools will require more than just infrastructure. If RNSs are to play a constructive role in facilitating collaboration, they will need to improve on the established method of using existing collegial contacts to find the “friend of a friend” who might provide needed expertise. To do this, they will need to provide easy access to high-quality data; in effect, they must provide added value unavailable through other means [6]. Furthermore, they must support the potentially different goals of different groups of users [12].

Although previous efforts have investigated collaborator search habits and preferences, relatively little attention has been paid to how interactive tools might meet these needs. Investigation of potential features and how they might be realized addressed requirements identified in earlier studies, including the importance of personal contact lists [6] and geographic location [14], along with others that were implied, if not explicitly discussed, such as the temporal histories of grants and publications. In contrast with computational methods that attempt to model researcher similarity [19,20], the designs considered in this study rely on term matching and visual displays, thus favoring clarity and simplicity at the potential expense of missing latent similarities. Further comparisons of this tradeoff might be an interesting area for future investigation.

Participants in the qualitative inquiries did not respond enthusiastically to some features that were identified as potentially important in prior work [5,6]. In response to the prototype based on personal social networks, participants were not particularly interested either in the use of their personal contacts as seed points or in the use of geographical distance as a criterion for selecting collaborators. However, in both cases participants may have missed the salient point. In the case of personal social networks, participants’ reaction that “they know these people already” may have overshadowed the fact that existing colleagues are important “gateways” to people they do not know. In the case of geographical distance, participants may not have been aware of the potential impact of proximity on collaborative productivity and of the possibility of discovering neighboring, but unknown, collaborators. Whatever the etiology, these findings suggest the likelihood of a range of preferences and styles for searching for collaborators. More fully realized tools might provide users with a range of starting points, views, and filtering options.

Positive responses to the prototype suggest the design provided useful functionality for collaborator searches. Participants found the timeline-based display of publications and grants to be useful for a variety of tasks, including identifying central people in fields, assessing researchers’ levels of activity and finding multidisciplinary collaborators. Timeline displays of publication activity have also been explored in other RNSs, most notably Profiles[15] and SciVal [16].

Participants in the first phase of the study were inconsistent in their comments regarding the role of impact measures in collaboration search processes. Although these metrics were generally found to be of potential use, there was little agreement on which specific measures might prove most useful. It is possible that this lack of consensus is a reflection of the ongoing discussion of the relative merits of different measures [41,42]. Potential design solutions might include displaying multiple impact measures, along with tools for filtering and ranking along individual measures or potentially some weighted aggregate measure.

The results for the SUS present both initial feedback on the usability of the functional prototype and indications of areas potentially in need of further work. The mean SUS score of 76.4 (SD 13.9) provides some validation of the usability of the tool, with particularly encouraging scores for questions regarding ease of learnability and confidence in using the system. Other questions suggest potential concerns regarding unnecessary complexity, potential need for technical support, inconsistency, and the need for training.

A relatively low score for the question involving frequency of use (“I think that I would like to use this system frequently”) is consistent with earlier observation that most researchers do not use online tools to find collaborators (see Table 4) and with the observation that finding collaborators may not be seen as a discrete or frequent task. Further study including empirical comparisons of metrics, such as learnability, would be needed to better understand these preliminary usability results.

Additional Likert questions assessing satisfaction with specific design elements gave generally encouraging results. The lowest score was given to the representation of the documents within the system (“height of the publication bar”), which scored 2.90. During the design phase of the working prototype, several approaches for representing the documents were considered. The initial design suggestion was to use absolute scales making the height of each bar proportional to the number of publications by a candidate that matched the keyword in each given year. This approach was rejected initially because it complicated rendering for candidates with keywords or bars that would contain low but nonzero counts. Furthermore, absolute counts might perpetuate biases against junior researchers, who might be less likely to have many publications matching a single topic in any given year.

Instead, we used a relative scaling approach normalizing the height of each bar to the percentage of that individual’s publications on the given topic for the given year. This design presents its own challenges because researchers with similar ratios but vastly different outputs on a given topic could be represented identically. Alternative representations with appropriate user controls might give users the option of selecting a preferred visual representation and further comparative user testing might be needed to better understand the usability implications of these different layouts.

Questions regarding initial design elements also provide preliminary validation of the requirements identified in the qualitative investigation of the paper prototypes (Table 6). Positive responses to the timeline (requirement 1), the list of potential collaborators (requirement 3), and the multiple keyword search (requirement 4) suggest that these features might play important roles in production-quality collaborator search tools. However, the current list of requirements and themes is not definitive. Further exploration of user needs, involving a broader set of informants, is likely necessary to capture the possible variations in preferences and working styles for collaboration identification.

These inquiries identified several additional suggested features focusing on the presentation of richer information about potential collaborations. The addition of academic degrees, impact factors, and research resources [38-40] might provide additional perspective on the prominence of potential collaborators. Exploration of the relative utility of these comparative measures might be an interesting focus for future work.

Participant recruitment identified subpopulations of users with potentially different needs and goals for research collaborator search tools. Because recruitment involved a convenience sample [43] based on email solicitations to scientists interested in research networks and subsequent snowball sampling, participants are in no way representative. It is entirely possible that this convenience sample might have introduced biases in the results.

However, we did identify 2 distinct groups with different goals and perspectives. Although the nature of the sample limits generalizations that might be made, PIs appeared to rely more heavily on personal networks than RFs (Table 4). Because the facilitators are generally working on behalf of others, potentially in unfamiliar fields, they might benefit more from interactive tools. Other potential features, such as concept maps relating topics from different fields, might provide additional benefits for research facilitators.

Participants also suggested that contextual differences might make interactive tools more useful to certain classes of PIs. Researchers at institutions that lack opportunities for local collaborations and junior researchers, previously described as relatively impoverished with respect to personal networks of potential collaborators [5], may be those who stand to benefit most from research social network collaboration identification tools.

The concern that collaborator search is not a discrete task that users engage in is consistent with the observation that search engines may lead many users into RNS pages [11]. To be successful, collaborator search tools will have to work within this well-established dynamic, finding ways to engage users who arrive via search engines and providing value beyond simple ranked lists. The functional prototype provides an initial design exploration that might move in this direction, but additional work will be needed to fully integrate this vision within the context of functional RNSs.

Further work will be needed to develop a more complete understanding of the use of collaboration search tools. The small and nonrepresentative sample of participants limits the breadth, depth, and generality of these preliminary results. Specifically, this study does not address the very real possibility that collaboration search practices and preferences may differ across the wide range of biomedical research collaborations. Differences in researcher backgrounds (basic researchers vs clinical researchers), number of collaborations, size of collaborations, local funding climate and incentives, and the extent to which research is interdisciplinary are just a few of the factors that might influence how researchers might identify potential collaborators and, therefore, how interactive tools might best support this practice.

### Limitations

This project’s small sample size limits the generalizability of the results. The convenience sample of 38 participants may not be representative of the greater research community. Generality of the results might also be limited by the diversity of the participant pool, which contained a relatively small number of researchers with medical degrees. Descriptions of collaborator search behavior are limited by reliance on recall-based measures and respondents’ definitions of the nature and extent of their collaborations. The limitations of the data used in the functional prototype (2 VIVO datasets) might have influenced users’ responses to the tool.

### Conclusions

The landscape of RNSs continues to evolve as more systems are deployed throughout institutions providing researchers novel opportunities for scientific collaborations. RNSs have the potential to play an important role in enabling interdisciplinary science. However, these benefits will not be realized without highly usable and useful end-user applications. Successful collaboration support tools must provide enough value to convince researchers to change established habits, including traditional networking and Web searches. Effectively converting the previously manual and socially complex task of identifying collaborators into a computer search system requires analysis of user needs and how tools might change/impact their workflow.

This qualitative study used semistructured interviews with researchers to gauge responses to paper prototypes for collaboration search tools. This inquiry identified 2 distinct user groups (RFs and PIs), and 3 themes categorizing collaboration search software needs: measure impact, track candidates, and conduct complex searches. Four specific requirements—chronological display of research output, robust impact measures, tools for tracking promising candidates, and multiple keyword searches—were considered for inclusion in a functional prototype, which was reviewed by participants in a second round of qualitative inquiry. Responses on the SUS provided initial formative validation of the design.

Although further inquiry will be needed to understand the similarities and differences between these subgroups, these distinctions illustrate the importance of understanding user needs and of providing functionality that meets those needs.

## Multimedia Appendix 1

Semi-structured interview questions used in Phase 1.

## Multimedia Appendix 2

Description: Semi-structured interview instrument for Phase 2 evaluation of functional prototype.

## Abbreviations

MeSH: Medical Subject Headings
NIH: National Institutes of Health
PI: principal investigator
RDF: Resource Definition Framework
RF: research facilitator
RNS: research networking system
SPARQL: SPARQL Protocol and RDF Query Language
SUS: System Usability Scale
