# Supplementary material for: Finding Collaborators: Toward Interactive Discovery Tools for Research Network Systems
Source: J Med Internet Res. 2014 Nov 4;16(11):e244. doi: 10.2196/jmir.3444 (PMC4376239; doi:10.2196/jmir.3444)
Supplement: Supplementary file 1 [file jmir_v16i11e244_app1.pdf]

Supplemental Appendix S1
Title: Semi-structured interview questions used in Phase 1
Legend: Semi-structured interview questions used in Phase 1

Description:.

1. Please indicate your experience level (student, post-doc, or faculty rank):
   a. If Faculty, please indicate your length of tenure:

2. How many funded research projects have you participated in during the past twelve months?

3. What was the average number of collaborators you directly interact with in each project?

4. What is the total number of unique collaborators you have interacted with in the last twelve months?

5. During the past 12 months, how many times have you been approached for a collaboration?

6. During the past 12 months, how many times have you contacted someone about a collaboration?

7. How do you usually find collaborators?

8. Are there specific tools you use to find collaborators (e.g. PubMed, NIHReporter) ?

9. Do you use general purpose networking applications (e.g. FaceBook, LinkedIn, Google+, etc.)?

   If so:

   a. How often do you use the application?

   b. Have you used this application to find a collaborator?

   c. If so, were you successful?

   d. What changes would make this application easier to find collaborators?

10. Do you use a scientific collaboration tool (e.g. VIVO, CAP, Loki, ResearchGate, etc.)?

    If so:

    a. Is this tool provided by your institution?

    b. How often do you use the tool?

    c. Have you used this application to find a collaborator?

    d. If so, were you successful?

    e. What changes would make this application easier to find collaborators?

11. What is your workflow for searching for a collaborator?
