# Supplementary material for: Finding Collaborators: Toward Interactive Discovery Tools for Research Network Systems
Source: J Med Internet Res. 2014 Nov 4;16(11):e244. doi: 10.2196/jmir.3444 (PMC4376239; doi:10.2196/jmir.3444)
Supplement: Supplementary file 2 [file jmir_v16i11e244_app2.pdf]

Supplemental Appendix S2

Title: Description: Semi-structured interview instrument for Phase 2 evaluation of functional prototype.

Interview Questions

1. Please indicate your experience level (ex: student, post-doc, or faculty rank):
   a. If Faculty, please indicate how long you have been a faculty member:

2. Approximately, how many funded research projects have you participated in during the past twelve months?

3. Approximately, what was the average number of collaborators you directly interact with in each project?

4. What is the total number of unique collaborators you have interacted with in the last twelve months?

5. During the past 12 months, how many times have you been approached for a formal collaboration?

6. During the past 12 months, how many times have you contacted someone about a potential collaboration?

7. How do you usually find collaborators?

8. How do collaborators find you?

9. Are there specific tools you use to find collaborators (e.g. PubMed, NIHReporter, Web of Science) ?

10. Do you use general purpose networking applications for professional purposes (e.g. FaceBook, LinkedIn, Google+, etc.)?
    If so:
    a. How often do you use the application?
    b. Have you used this application to find a collaborator?
    c. If so, were you successful?
    d. What changes would make this application easier to find collaborators?

11. Do you use a scientific collaboration tool (e.g. VIVO, CAP, Loki, ResearchGate, etc.)?
    If so:
    a. Is this tool provided by your institution?

b. How often do you use the tool?

c. Have you used this application to find a collaborator?

d. If so, were you successful?

e. What changes would make this application easier to find collaborators?

12. What steps do you take when searching for a collaborator?

13. Describe a time when you successfully found a collaborator

Ask the participant to perform the two tasks.

After the tasks are completed:

Usability Metrics – Systems Usability Scale (Brooke 1996)

		**Strongly disagree**				**Strongly agree**
	**Question**	**1**	**2**	**3**	**4**	**5**
1	I think that I would like to use this system frequently					
2	I found the system unnecessarily complex					
3	I thought the system was easy to use					
4	I think that I would need the support of a technical person to be able to use this system					
5	I found the various functions in this system to be well integrated					

6	I thought there was too much inconsistency in this system					
7	I would imagine that most people would learn to use this system very quickly					
8	I found the system very cumbersome to use					
9	I felt very confident using the system					
10	I needed to learn a lot of things before I could get going with this system					

Evaluation Questions

1. What characteristics about a collaboration do you utilize when searching for a collaborator? For example, if you need someone to help you get a grant versus another person to co-author a paper, do you look for different information about them?

2. What additional data would you like to know about potential collaborators?

3. What additional data would you like to provide to people seeking you as a collaborator?

4. Are there other applications you would suggest connecting to this application (e.g. social networking, online bibliometric tools, etc.)?

5. What features would you suggest adding to the application?

6. If you had to pay to use this application, how much would you be willing to pay?

7. On a scale of 1-5 (1 being useless and 5 being most useful) Please rate the utility the features found in the prototype:

		**Useless**				**Useful**
	**Question**	**1**	**2**	**3**	**4**	**5**
1	Timeline format					
2	Heights of doc rectangle percentage of keywords per year					
3	Document data available on hover					
4	Grant title and grant role					
5	Grant start/end dates					
6	Ability to add/remove authors to a followup list					
7	Recording notes about the authors on the followup list					
8	Ability to add/remove additional keywords					

An explanation of the prototype functionality rated using Likert-type questions.

- **Overall timeline format**: The participants were asked to rate the utility of arranging the publication and grant data along a chronological axis.
- **Height of the publication bar**: The height of the publication bar corresponded to the percentage of a candidate's publications in a given year that matched a specified keyword. For example, a rectangle representing 6 "Alzheimer disease" publications out of 10 publications would have a height equal to 60% of the overall height of the vertical region for the candidate.
- **Document data available on hover**: The user could hover their mouse over one of the document rectangles and see a list of: the number of articles for the topic and the titles of the publications.

- **Grant title and role available on hover**: The prototype allowed the user to hover over the end points of the grant (represented by green triangles). During a hover event, the user saw: the grant title and the candidate's role in the grant (principal investigator, co-investigator, etc.).
- **Grant start and end dates indicated on timeline**: Green triangles indicate the grant start and end dates. Hovering one of the triangles draws a green line to its corresponding triangle indicating the overall length of the grant. For example, hovering over the end date of a grant would display a green line connecting it to the start date for that grant.
- **Ability to add/remove collaborators to a list**: The prototype introduces a concept of a "followup list" allowing the users to add/remove candidates to a list. This list can be utilized later when attempting to contact the most promising candidates.
- **Ability to add notes regarding candidates**: The followup list had additional functionality allowing users to record observations regarding candidates. For example, a user could add a candidate to the followup list and attach a note "Dr. XYZ can provide an introduction to this candidate."
- **Ability to add/remove additional keywords**: A user will search first using an initial keyword entered in the textbox on the upper left of the page. The screen returns the initial list of candidates and a list of additional keywords as checkboxes. A user can leverage these additional keywords displaying more publications related to the initial keyword.

References

Brooke J. 1996. SUS-A quick and dirty usability scale. *Usability evaluation in industry* 189:194.
